# In Vitro Antiviral Activity of a Silydianin-Rich Extract from *Silybum marianum* Seeds Against Four Strains of Enteroviruses: EV71, Coxsackievirus B2, Coxsackievirus A10, and Poliovirus SL-1 and Its Impact on Improving Delayed Gastric Emptying in Mice

**DOI:** 10.3390/antibiotics14020196

**Published:** 2025-02-14

**Authors:** Houda Zaher, José Francisco Quílez del Moral, Sanae Lemrabet, Neri Koutchala, Bouchaib Bencharki

**Affiliations:** 1 Laboratory of Agro-Alimentary and Health, Faculty of Sciences and Techniques, Hassan First University of Settat, Settat 26000, Morocco; bouchaib.bencharki@uhp.ac.ma; 2 Department of Organic Chemistry, Institute of Biotechnology, University of Granada, 18071 Granada, Spain; jfquilez@ugr.es; 3 Virology Department, National Institute of Hygiene, Ministry of Health, Rabat 10020, Morocco; sanae.lem@gmail.com; 4 Department of Computer Science and Artificial Intelligence, Technical School of Computer Engineering and Telecommunications, University of Granada, 18071 Granada, Spain; e.nerik@go.ugr.es

**Keywords:** gastroparesis, enteroviral infections, silydianin, *Silybum marianum*, antiviral activity, gastrointestinal transit

## Abstract

Background: Gastroparesis, a chronic digestive disorder characterized by delayed gastric emptying, often results from diabetes, post-surgical complications, autoimmune diseases, and neurological disorders. In approximately 50% of cases, the cause is idiopathic gastroparesis (IGD). Recent studies suggest a link between chronic enteroviral infection and persistent gastrointestinal symptoms, including delayed gastric emptying. This study investigates the effects of a silydianin-rich extract from *Silybum marianum* seeds on enteroviral infections in vitro and the mitigation of delayed gastric emptying in mice. Silydianin, a key bioactive compound known for its liver-protective and antioxidant properties, has not been extensively studied for its impact on enteroviral infections and gastroparesis. Methods: NMR spectroscopy (^1^H, ^13^C, DEPT 135 and 2D, and HSQC) and HRMS identified silydianin as the primary compound, with minor flavonolignans. This study assessed the cytotoxicity and antiviral activity of the extract at various stages of the viral life cycle, including virucidal activity, cell protection, and post-infection effects, using neutral red assays in RD cells, with results confirmed by real-time PCR. The viruses studied included coxsackievirus B2, coxsackievirus A10, poliovirus SL-1, and enterovirus EV71. The impact on delayed gastric emptying was evaluated in a mouse model using doses of 100 and 200 mg/kg compared to a control group receiving physiological saline. Results: The silydianin-rich extract showed consistent antiviral activity, with the highest selectivity index (SI) for EV71 (4.08) during virucidal activity. It provided moderate cell protection, with EC_50_ values ranging from 120.88 to 186.10 µg/mL and SI values from 2.20 to 3.39. Post-infection treatment showed varying efficacy, with coxsackie A10 demonstrating the highest SI (3.90). In vivo, the extract at 200 mg/kg significantly improved gastric emptying to 96.47% and slightly increased gastrointestinal transit from 50.33% to 61.46%. Conclusions: These results suggest that silydianin may be effective for treating enteroviral infections and enhancing intestinal function, making it a promising candidate for gastroparesis treatment and warranting further research.

## 1. Introduction

Gastroparesis is a complex disorder characterized by delayed gastric emptying without an obvious mechanical obstruction, leading to a range of debilitating symptoms such as nausea, vomiting, bloating, and abdominal pain [1]. The etiology of gastroparesis remains poorly understood, with approximately 50% of cases classified as idiopathic. Other cases are associated with conditions like diabetes, post-surgical complications, autoimmune diseases, and neurological disorders [2]. Recent research suggests a potential link between enteroviral infections and persistent gastrointestinal symptoms, including delayed gastric emptying [3].

The frequency of enterovirus infections and the diseases they cause vary according to serotype, geography, season, host age, and antibody status [4,5]. Enteroviruses are a diverse family of non-enveloped small RNA viruses known to affect the gastrointestinal tract and may contribute to ongoing symptoms even after the acute phase of infection has resolved [5]. Within the family Picornaviridae, the enterovirus group comprises 12 species. Enterovirus A species includes 25 serotypes, among which are those responsible for hand, foot, and mouth disease (HFMD) such as EV-A71, CV-A16, CV-A5, CV-A6, CV-A8, and CV-A10 [6]. The enterovirus B species includes coxsackieviruses B1-B6 (CVB1-B6), coxsackievirus A9 (CVA9), and more than 30 serotypes of echoviruses. Polioviruses are classified into three types: PV1, PV2, and PV3 [7].

Enteroviruses are responsible for a broad spectrum of human diseases, ranging from severe, permanent paralysis to mild febrile illnesses. While some enteroviruses frequently cause epidemics associated with specific syndromes, the same serotypes can lead to sporadic infections with diverse clinical manifestations or remain asymptomatic in different contexts, making clinical diagnosis alone unreliable [8].

Coxsackieviruses, a subgroup of enteroviruses, are linked to various illnesses such as aseptic meningitis, herpangina, epidemic myalgia (also known as Bornholm disease), hand, foot, and mouth disease (HFMD), myocarditis, pericarditis, pneumonia, rashes, and the common cold. They may also contribute to congenital malformations and potentially certain forms of diabetes [8]. Coxsackievirus B2, in particular, can cause chronic enterovirus infections of the heart, leading to congestive heart failure, for which heart transplantation may be the only treatment option [9]. The introduction of reverse transcription polymerase chain reaction (RT-PCR) as a diagnostic tool for suspected myocarditis underscores the urgent need for effective antiviral treatments [9].

Enterovirus EV71 and coxsackievirus A10 are major etiological agents of HFMD. EV71 is particularly concerning, as it constitutes 80–85% of pathogens isolated from HFMD-related deaths, according to clinical etiological data [6]. EV71 has become a focus for WHO surveillance in the post-polio eradication era, most frequently affecting children. It can be asymptomatic or lead to severe neurological disease [6].

Among enteroviruses, polioviruses can cause the most severe diseases, such as paralytic poliomyelitis. Poliovirus infection progresses to poliomyelitis when the virus invades the central nervous system, attacking the motor neurons in the spinal cord. The result is muscle weakness, generalized paralysis, and, eventually, death, usually due to respiratory muscle failure [10]. Since the launch of the global polio eradication initiative (GPEI) in 1988, the number of paralytic cases linked to wild poliovirus (WPV) has decreased from 350,000 in 1988 to just six cases in 2023, five cases in Afghanistan, and one case in Pakistan [11]. WPV2 and WPV3 were last detected in 1999 and 2012, respectively, leaving WPV1 as the only remaining endemic strain [12].

The oral polio vaccine (OPV) is a live-attenuated vaccine containing weakened strains of the poliovirus, administered orally. In rare cases, the weakened virus in OPV can revert to a virulent form, leading to vaccine-associated paralytic poliomyelitis (VAPP) [11]. This occurs when the attenuated virus replicates in the intestines and migrates to the nervous system, causing paralysis similar to that caused by wild poliovirus [11].

Surveillance remains the primary strategy to control these diseases while waiting for the development of novel antiviral therapies. Researchers are increasingly focusing on natural products as potential treatments due to their safety profiles and availability for trials.

Flavonolignans are a notable class of secondary plant metabolites formed through the oxidative coupling of a flavonoid and a phenylpropanoid component [13]. These compounds have demonstrated a variety of biological activities, including therapeutic properties for gastrointestinal conditions [14]. The most well-known source of flavonolignans is the seed extract of *Silybum marianum* (milk thistle), commonly referred to as silymarin. Silymarin primarily contains seven flavonolignans: silybin A, silybin B, isosilybin A, isosilybin B, silychristin, isosilychristin, and silydianin [13]. Among plant extracts, silymarin is one of the most extensively studied for its mechanisms of action, particularly in the treatment of liver diseases [15]. It has been widely used in modern therapy for managing liver conditions and providing antioxidant effects [14]. Silybin, a key component of silymarin, has demonstrated significant antiviral activity against the *hepatitis C* virus, particularly when administered intravenously in clinical trials, and was well tolerated by patients [16].

Silydianin, a key bioactive compound (5–10%) found in silymarin extracted from *Silybum marianum* seeds, is known for its role in treating liver fibrosis and protecting the liver from toxins, and it is commonly used as a dietary supplement [17]. However, its potential antiviral and gastrointestinal benefits, particularly in relation to enteroviral infections and gastroparesis, have been largely underexplored. Research on silydianin’s chemistry and biological properties is limited, with scientists citing challenges in its isolation and purification, which restricts its availability for further study [17].

On the other hand, a study conducted on mice using intraperitoneal administration of 100–200 mg/kg of silibinin, silymarin, and taxifolin demonstrated a significant reduction in intestinal transit by 23–41% (*p* ≤ 0.5–0.01). This inhibitory effect on intestinal function is mediated through α-adrenergic and calcium pathways, indicating that these compounds may be effective as anti-diarrheal agents [18]. Additionally, silymarin has demonstrated a protective effect in a mouse model of necrotizing enterocolitis (NEC), significantly reducing free radical levels and oxidative stress and improving the severity of intestinal damage caused by NEC [19]. NEC is a serious gastrointestinal disease that primarily affects premature infants or newborns with low birth weight. Given that silydianin is a key component of silymarin extract, it may offer similar protective effects against intestinal damage caused by enteroviral infections. This suggests the potential for silydianin to improve intestinal function, as demonstrated in this study’s tests on normal mice and supports further research into its effectiveness in treating enterovirus-induced gastroparesis in mice. The use of a mouse model in our study is based on its well-established relevance to human disease conditions, particularly in gastrointestinal research. Mice share key physiological and genetic similarities with humans, making them an effective model for studying gastroparesis and other gastric motility disorders [20]. Mouse models of obesity and diabetes are commonly employed to investigate the relationship between metabolic disorders and delayed gastric emptying, supporting the translatability of our findings to human conditions. Studies using these models have provided crucial insights into the mechanisms underlying gastric motility disorders and have been instrumental in testing prokinetic agents to restore normal gastric function and improve glycemic control [21]. For instance, young ob/ob mice, known for rapid gastric emptying, overeating, and weight gain, highlight the significant role of proper gastric motility in metabolic disorders and comorbidities often seen in humans with gastroparesis [21]. Additionally, various studies in mouse models of delayed gastric emptying have demonstrated their ability to mirror human gastric motility disorders, providing valuable insights into human gastric physiology and pathology [21,22,23,24].

In this study, we explore the potential of a silydianin-rich extract from *Silybum marianum* seeds to inhibit various stages of the viral life cycle, including virucidal activity, cell protection, and post-infection effects. Using a cytopathic effect assay confirmed by real-time PCR, we aim to evaluate the efficacy of the extract against coxsackievirus B2, coxsackievirus A10, poliovirus SL-1, and enterovirus EV71 in vitro. Additionally, we assess its impact on delayed gastric emptying (gastroparesis) in mice in vivo by pretreatment for 7 days. Through this research, we seek to provide valuable insights into the therapeutic potential of silydianin, particularly in treating chronic enteroviral infections and associated gastrointestinal disorders.

## 2. Results

### 2.1. Characterization of Silybum marianum Extract Using NMR Spectroscopy

**Figure 1:**
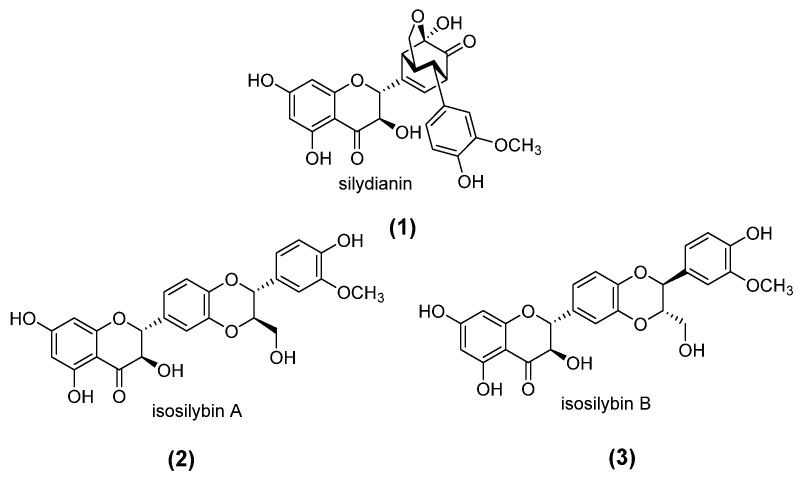
Structures of flavonolignans **1**–**3**.

**Figure 2:**
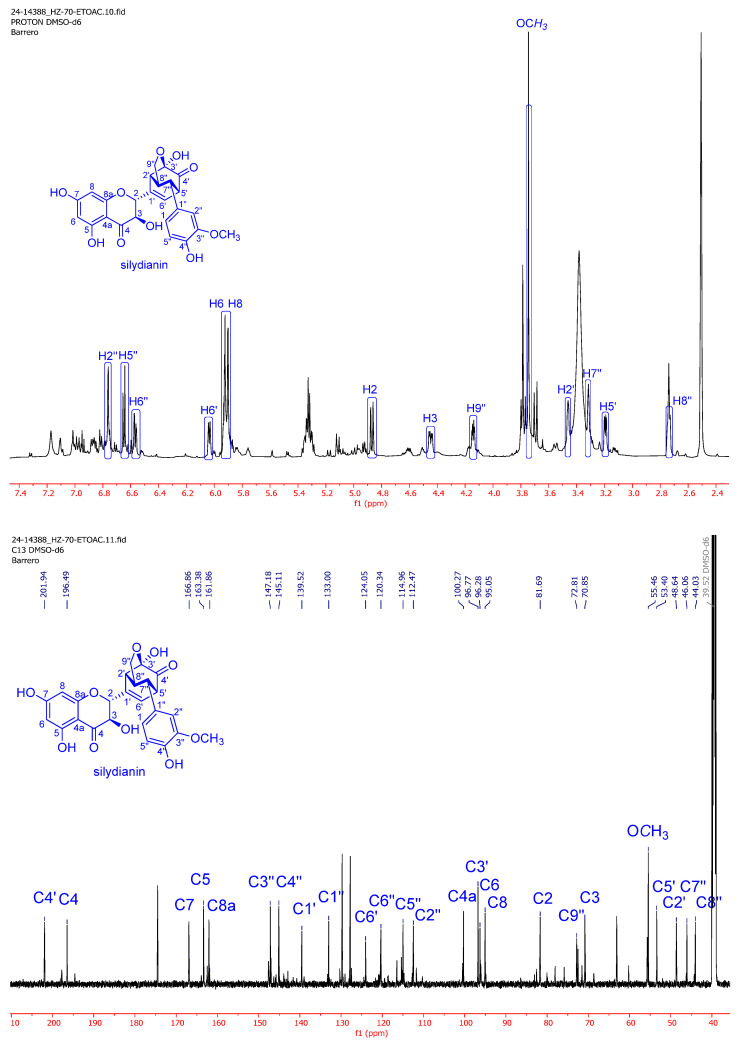

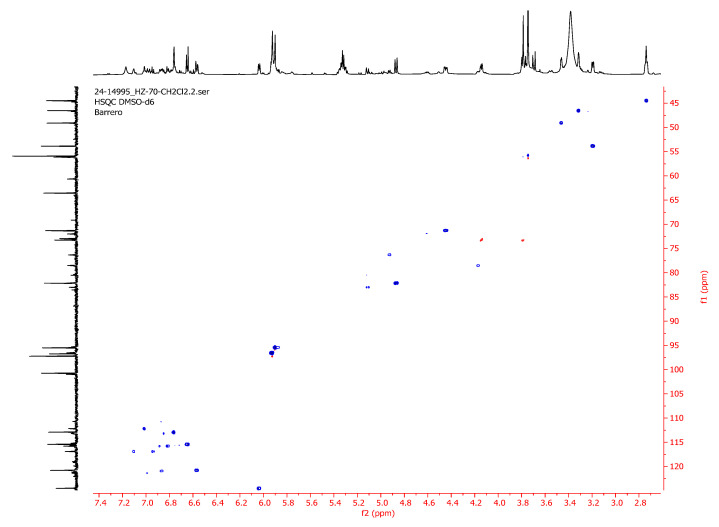
Silydianin signal assignments in the NMR spectra of the EtOAc extract of *S. marianum*.

The seed extract was fractionated into three fractions using ethyl acetate (EtOAc), chloroform (CHCl_3_), and *n*-butanol (*n*-BuOH). NMR spectra of the EtOAc and CHCl_3_ fractions showed that silydianin (**1**) (Figure 1) was the predominant compound in these extracts, while the *n*-BuOH fraction contained primarily sugars. The identification of silydianin was achieved after comparing its spectroscopical data—including NMR (Figure 2) and HRMS ([M+H]^+^ m/z 481.1130, calculated for C_25_H_21_O_1_, 481.1135)—with those published for this compound [25,26]. In addition to silydianin, NMR spectra of the extract showed signals compatible with the presence of other flavonolignans, such as isosilybin A (**2**) and isosilybin B (**3**) [25,26].

### 2.2. Cytotoxicity of Silybum marianum Extract on RD Cells

**Figure 3:**
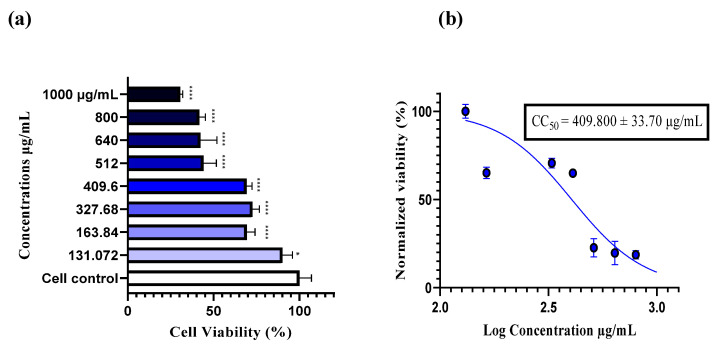
Dose-dependent effects of hydro-methanolic extract on RD cell viability. (**a**) Viability of RD cells treated with various concentrations of the extract (ranging from 131.072 to 1000 µg/mL) following 48 h of incubation. Cytotoxicity was assessed using the neutral red assay, and absorbance was measured with a microplate reader at 540 nm ± 10 nm. Statistically significant differences from the control cells are indicated as * *p* < 0.05, **** *p* < 0.0001, as determined by one-way ANOVA followed by Tukey’s post hoc test. Data are expressed as the mean ± S.E.M. (**b**) The CC_50_ value was determined by applying a non-linear regression curve to the normalized data. The CC_50_ is presented as the mean ± SD.

The cytotoxicity of the plant extract was evaluated using a neutral red assay. A stock solution of the extract was prepared at a concentration of 2 mg/mL in DMSO, which showed no cytotoxic effect at a concentration of 1%. Subsequently, a range of concentrations was prepared in DMEM (0% FBS), ranging from 1000 to 131.072 µg/mL. After incubation at 37 °C for 48 h, the results showed a significant dose-dependent decrease in cell viability (**** *p* < 0.0001). At 131.072 µg/mL, 90% cell viability was recorded, indicating this as the safest concentration within the tested range, while at 1000 µg/mL, the highest cytotoxic concentration was observed, with only 30% cell viability. The half maximal cytotoxic concentration (CC_50_) was determined to be 409.800 µg/mL, indicating that 50% of the cells remained viable at this concentration. Based on these results, four high concentrations that showed minimal or no cytotoxicity were selected for antiviral assays: 400, 200, 100, and 50 µg/mL (Figure 3).

### 2.3. The Antiviral Assays by the Neutral Red Uptake

**Figure 4:**
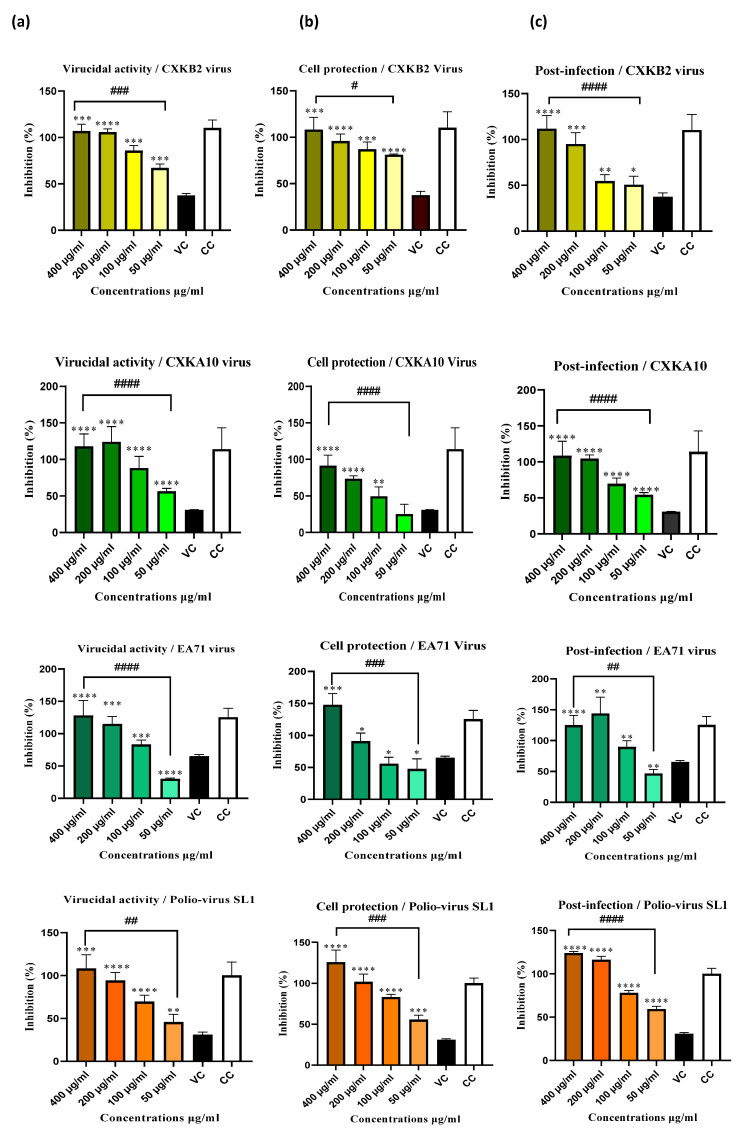
*Silybum marianum* extract exhibited dose-dependent viral inhibition against four enteroviruses: coxsackie B2, coxsackie A10, EA71, and poliovirus SL-1. Viral inhibition was evaluated using the neutral red uptake assay in three different experiments: (**a**) virucidal assay, (**b**) cell protection assay, and (**c**) post-infection assay. Data are presented as mean ± S.E.M., with error bars representing the range of values from three independent experiments. Statistical significance compared to the virus control (VC) was analyzed using one-way ANOVA followed by Tukey’s post hoc test, with significance levels indicated as * *p* < 0.05, ** *p* < 0.01, *** *p* < 0.001, and **** *p* < 0.0001. Additionally, significant inhibition as a function of concentration is indicated by # *p* < 0.05, ## *p* < 0.01, ### *p* < 0.001, and #### *p* < 0.0001.

**Figure 5:**
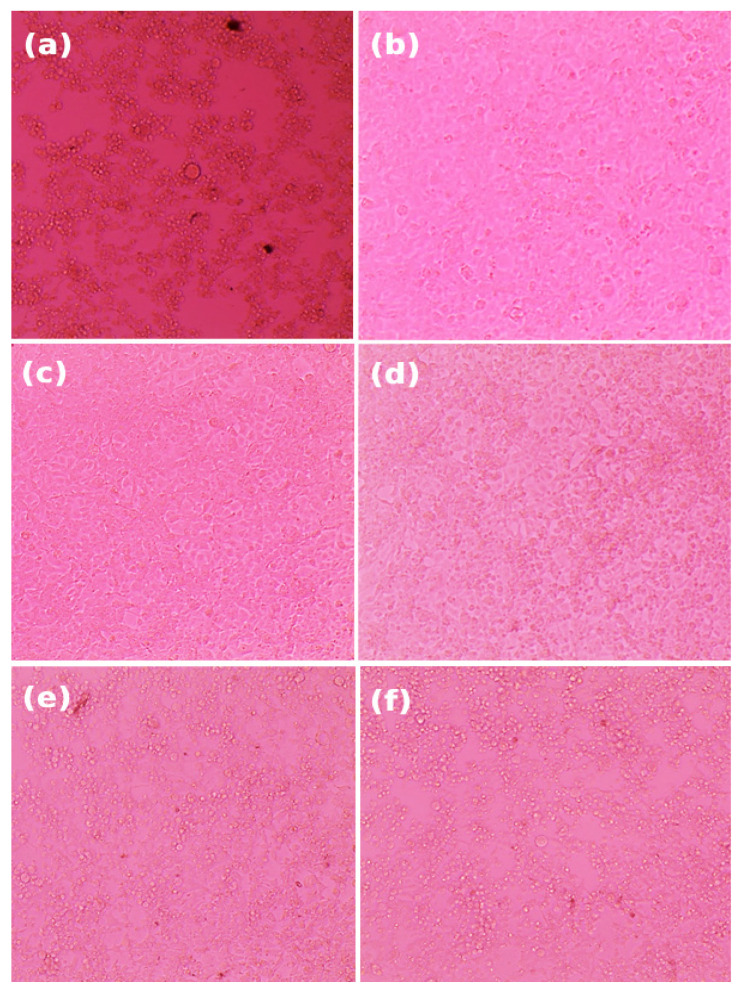
The monolayers were microscopically examined for viral cytopathic effects (CPE) in a dose-dependent manner, focusing on cell rounding and other significant morphological changes compared to control cells after a 48 ± 2 h incubation with the treatment concentrations prior to performing the neutral red assay. (**a**) Virus control; (**b**) cell control; (**c**) treatment with 400 µg/mL; (**d**) treatment with 200 µg/mL; (**e**) treatment with 100 µg/mL; and (**f**) treatment with 50 µg/mL.

**Figure 6:**
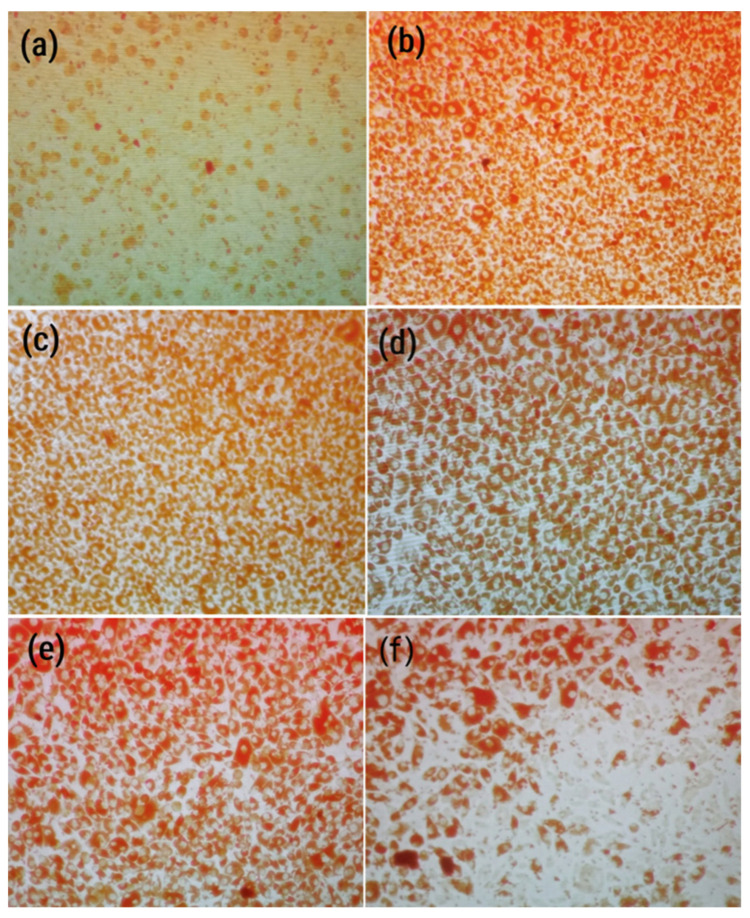
Cell viability stained by neutral red following the neutral red method (as detailed in the Methods section) after 48 h of incubation with virus and treatment concentrations: (**a**) virus control, (**b**) cell control, (**c**) treatment with 400 µg/mL, (**d**) 200 µg/mL, (**e**) 100 µg/mL, and (**f**) 50 µg/mL.

The antiviral activity of *Silybum marianum* extract was assessed in three phases of infection: virucidal assay, cell protection, and post-infection against three common enterovirus strains typically found in children’s stool: enterovirus (EV)-71, coxsackie B2, coxsackie A10, and one vaccine strain of poliovirus (SL-1) that can sometimes convert to a pathogenic form, potentially causing acute flaccid paralysis in children. The absorbance of cell viability at 570 nm was measured using a microplate reader to evaluate the viral inhibition of infected cells incubated with extract doses (400, 200, 100, and 50 µg/mL) for 48 h, using the neutral red method. The results, shown in Figure 4, indicate a significant reduction in viral cytopathic effects in a dose-dependent manner. The highest concentration (400 µg/mL) exhibited the most substantial antiviral activity across all three points of infection, with treatment completely inhibiting viral effects (100% inhibition) for all tested viruses (Figure 5). The lowest concentration (50 µg/mL) also showed significant effects, achieving 80% cell protection and 60% extracellular virucidal effect against coxsackie B2, as well as approximately 55% inhibition of viral infectivity across all three infection phases against poliovirus (Figure 6). These results were statistically significant with ** *p* < 0.01, *** *p* < 0.001, and **** *p* < 0.0001. Using regression analysis, absorbance values were used to calculate the effective concentration for 50% viral inhibition (EC_50_) and the selectivity index (SI), expressed as the CC_50_/EC_50_ ratio, for the *Silybum marianum* extract against the four virus strains tested (Table 1). The extract demonstrates moderate virucidal activity against all four viruses, with the most effective action against EV71 (EC_50_ of 100.31 µg/mL) and the highest SI (4.08), indicating it is the most selective agent against this virus. In the cell protection phase, the extract shows different levels of protection, with coxsackie A10 having the lowest EC_50_ (120.88 µg/mL) and the highest SI (3.39), suggesting it can significantly shield host cells from viral damage. In the post-infection phase, the extract exhibits strong antiviral activity, particularly against coxsackie A10 and EV71, with the lowest EC_50_ values (105.06 ± 1.96 and 107.67 ± 6.04 µg/mL, respectively) and the highest SI values (3.90 and 3.82, respectively). This indicates that the extract is highly effective at safely inhibiting viral replication, even after the initial infection (Table 1).

### 2.4. Real-Time RT-PCR Assay

Real-time RT-PCR with generic primers was used to assess the antiviral efficacy of a plant extract across different concentrations (200, 100, and 50 µg/mL) against various RNA virus strains. The ΔCt values, representing the changes in viral RNA levels compared to untreated controls, provide insights into the degree of viral replication inhibition, with higher ΔCt values indicating stronger antiviral effects. The evaluation was conducted across three viral life cycle stages: virucidal activity, cellular protection, and post-infection.

The extract demonstrated substantial virucidal activity against EV71 across all concentrations, with the most pronounced effect at 200 µg/mL. For coxsackie B2, significant viral inhibition was observed at 200 µg/mL and 100 µg/mL. Remarkably, no detectable RNA was found for coxsackie A10 at any tested concentration, signifying highly effective viral inhibition. The extract also provided moderate cellular protection, particularly against coxsackie B2 and EV71, with significant protection at 200 µg/mL and 100 µg/mL. Notably, coxsackie A10 showed no detectable viral RNA at these concentrations, confirming the strong inhibition of viral replication. Even at 50 µg/mL, some protection was observed for coxsackie A10, with a ΔCt equal to 2.167 ± 0.707 in relation to the control virus.

In the post-infection phase, the extract exhibited robust antiviral activity against coxsackie B2 and EV71 at all concentrations, with the most notable inhibition at 200 µg/mL. It also displayed moderate to significant inhibition against coxsackie A10. Interestingly, no detectable RNA for poliovirus SL-1 was observed at any concentration or viral life cycle stage tested, underscoring the broad-spectrum antiviral potential of the extract (Table 2).

### 2.5. Effect of Extract on Gastric Emptying In Vivo

**Figure 7:**
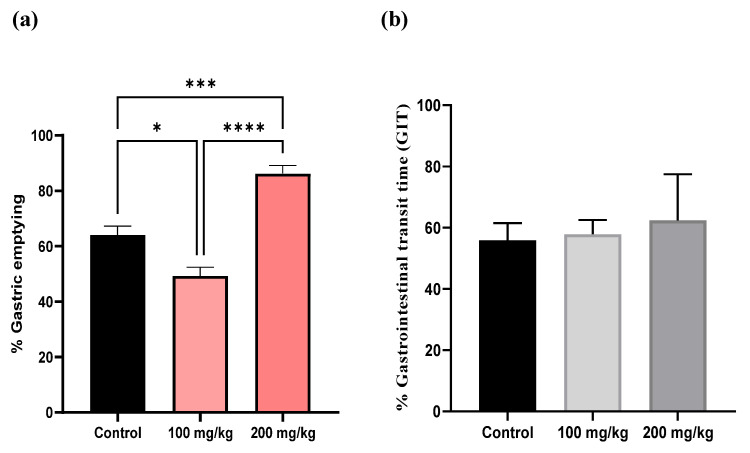
Impact of *Silybum marianum* extract on (**a**) % gastric emptying and (**b**) gastrointestinal transit time (GIT). The effects of *Silybum marianum* extract on gastric emptying were assessed using the phenol red method, with absorbance measured at 570 nm. Data are expressed as mean ± SEM (*n* = 6). Statistical analysis was performed using one-way ANOVA and *t*-tests for unpaired data, with significance levels denoted as * *p* < 0.05, *** *p* < 0.001, and **** *p* < 0.0001. The gastrointestinal transit time data are presented as percentages and were found to be statistically non-significant.

The impact of the extract on gastric emptying was evaluated at two concentrations (100 mg/kg and 200 mg/kg) and compared with a control group. In control mice, which received only physiological saline, gastric emptying after 20 min ranged from 56% to 72%, representing the baseline rate of gastric emptying. At a concentration of 100 mg/kg, the extract significantly reduced gastric emptying, with values ranging from 40.45% to 56.03% (* *p* < 0.05), indicating a delay in gastric emptying compared to the control. However, the gastrointestinal transit speed, which measures how quickly contents move through the intestines after leaving the stomach, did not differ significantly from baseline. Conversely, at 200 mg/kg, the gastric emptying percentages ranged from 79.78% to 96.47%, which are generally higher than those at 100 mg/kg (**** *p* < 0.0001) and in some cases exceed the control values (*** *p* < 0.001). At this higher concentration, gastrointestinal transit ranged from 50.33% to 61.46%, indicating a slight increase compared with the control. However, these differences were not statistically significant (Figure 7).

## 3. Discussion

Despite significant progress toward polio eradication, acute flaccid paralysis (AFP) continues to be a major public health concern, primarily due to the involvement of non-polio enteroviruses (NPEVs) and other pathogens like adenoviruses. These viruses are not only implicated in AFP but are also responsible for a variety of other diseases, including hand, foot, and mouth disease (HFMD), myocarditis, and viral meningitis. A study conducted by our laboratory, the national polio laboratory of Morocco (NPLM), during 2018–2019 revealed that 13% of AFP cases were associated with enteroviruses, while 8% showed adenovirus involvement. Notably, 2.2% of cases exhibited co-infections with both enteroviruses and adenoviruses [27]. The enteroviruses identified in these stool cases included EV71, coxsackie B2, and coxsackie A10 (unpublished data), which were subsequently used in our study on their antiviral activity. Similarly, another study confirmed the presence of NPEVs (12.7%) and adenoviruses (12.7%) in stool specimens from AFP cases [28]. However, it remains challenging to definitively identify the viral agent responsible for paralysis in many cases. These findings underline the critical need for continued virological surveillance of AFP worldwide and the urgent development of effective antivirals against enteroviruses to prevent them from causing more severe health issues in humans. Our investigation into the antiviral activity of flavolignans extracted from *Silybum marianum* against enteroviruses revealed promising results. The cytotoxicity on RD cells recorded after 48 h of incubation was 409.800 ± 33.70 µg/mL, indicating a higher CC_50_ compared to the 160.20 µg/mL reported by Lalani et al. [29] for RD cells. However, our findings are consistent with those of Fernando da Silva et al. [30] and Lalani et al. [31] in Vero cells, for which the CC_50_ values were 596.19 µg/mL and 425.1 µg/mL, respectively. This variation could be attributed to differences in the sources of silymarin, which was commercially provided in these studies, as well as differences in the cell lines used. Additionally, the *Silybum marianum* plant and its standardized extract, silymarin, are well-known for their safety profile. Silymarin has been shown to be well tolerated up to a lethal dose (LD_50_) of 10 g/kg in various animal models, and its oral administration has an established safety threshold of 1000 mg/kg body weight for two weeks [16]. Moreover, the acute toxicity of *Silybum marianum* fruit extract following intravenous administration was assessed in mice, rats, and dogs. The findings indicated that the single oral lethal dose (LD_50_) exceeded or was equal to 900 mg/kg in mice, 800 mg/kg in rats, and 300 mg/kg in dogs, demonstrating differential tolerance across these species [32]. This safety profile makes the plant and its compounds excellent candidates for widespread biological and health applications. Indeed, *Silybum marianum* has demonstrated potential activity in various infectious and non-infectious diseases, including notable antiviral properties. Commercial silymarin has shown promising effects, such as against the Mayaro virus (MAYV) post-infection, with a 50% effective concentration (EC_50_) of 3.58 μg/mL and a selectivity index (SI) of 29.6 [33]. In another study, silymarin exhibited an inhibitory effect on influenza A/PR/8/34 virus replication, where 100 μg/mL of silymarin inhibited late-stage viral RNA synthesis, comparable to oseltamivir [34]. Additionally, for Zika virus (ZIKV), which is associated with severe neurological syndromes and lacks a vaccine or treatment, silymarin demonstrated an EC_50_ of 34.17 μg/mL, with a selectivity index greater than 17 four times higher than that of the positive control, ribavirin [30]. In the post-entry stage of chikungunya virus (CHIKV) infection, silymarin showed an IC_50_ of 16.9 μg/mL with an SI of 25.1 [31]. Moreover, *Silybum marianum* extract exhibited inhibitory effects against hepatitis C virus (HCV) in both in vitro and in vivo studies by inhibiting HCV entry, RNA synthesis, viral protein expression, and infectious virus production [35]. This has led to significant interest in using the extract in large clinical trials for hepatitis infection and hepatoprotection [16].

Furthermore, silymarin has been tested against enterovirus 71 (EV71), alongside two other flavonoids, baicalein and baicalin, at various stages of the viral life cycle. Silymarin showed an extracellular virucidal effect with an IC_50_ of 15.2 ± 3.53 μg/mL and an SI of 10.53, but no inhibitory effect was observed in cell protection or post-infection activity [29]. These findings differ from our results, where the EC_50_ for virucidal activity was 100.31 ± 2.70 μg/mL, with significant effects on cell protection and post-infection stages, showing EC_50_ values of 186.10 ± 45.74 and 107.67 ± 6.04, respectively. This discrepancy could be attributed to differences in the silymarin tested. Commercial silymarin, known for being rich in silybin as the major component, may contain up to 39.2% silybins, 25.4% other flavonolignans, and 40% unknown components [36]. The studies listed above did not specify the chemical composition of the silymarin used. However, the literature suggests that silybin is a highly bioactive flavolignan, particularly known for its hepatoprotective and anti-HCV effects. Silybin has even been tested in humans with active viral hepatitis infection using a silybin–phosphatidylcholine complex called silipide for two months, resulting in a 10%–15% improvement in liver function [36]. It was also tested against the herpes simplex virus (HSV-2) with an IC_50_ of 100 μg/mL and a therapeutic index of 3.8 [37]. Silybin is widely recognized as the primary active component of silymarin and has been extensively studied in clinical trials. However, this focus on silybin may overlook the potential activities of other flavonolignan compounds. In our study, the extract was rich in silydianin (**1**), with minor quantities of isosilybins. This finding contrasts with previous reports [17], which found silydianin (**1**) to comprise 5–10% of silymarin extracts, but aligns with a study by Abouzid et al. in Egypt, which chemically classified two types of seed extract cultivars: silybin-rich and silydianin-rich. The silybin-rich extract was predominantly composed of silybin A, silybin B, and silychristin A, while the silydianin-rich extract was rich in silydianin (**1**), isosilychristin, and isosilybin (**2**–**3**). The correlations between these flavonolignans suggest shared biosynthetic pathways: silychristin A and silybins A and B are derived from the 4′-O-taxifolin radical, whereas silydianin (**1**), isosilychristin, and isosilybin B (**3**) originate from different flavonoid radicals [13]. The flavonolignan nucleus generally consists of the dihydroflavonol taxifolin linked to a coniferyl alcohol moiety through an oxeran ring (Figure 1). According to Chackalamannil et al. [38], this oxeran ring is crucial for the biological activity of these compounds, with the loss of this ring resulting in a loss of activity. Notably, only silybin and isosilybin possess this characteristic ring in the form of a 1,4-dioxane ring, which is key to their bioactivity [38]. On the other hand, silydianin (**1**) possesses a unique structure that sets it apart from other flavonolignans, contributing to its distinctive properties. While it shares a similar biosynthetic pathway with other silymarin flavonolignans, the key difference lies in its formation: silydianin (**1**) forms two new carbon–carbon (C-C) bonds, creating a unique bicyclic structure [17]. Additionally, the primary alcohol group of coniferyl alcohol undergoes intramolecular hemiacetalization, resulting in an unusual geminal ketone–hemiacetal structure (Figure 1) [17]. The oxidation product of silydianin, 2,3-dehydrosilydianin, characterized by a 2,3 double bond, has been shown to be more active than silybin, particularly in anticancer and antioxidant activities [39]. Structure–activity relationship studies suggest that the presence of a 2,3 double bond or phenolic hydroxyl group enhances biological activity [39]. Notably, the biological activity studies of silydianin (**1**) are limited [17], and its antiviral potential, particularly against the enteroviruses studied, has not been explored before. Returning to our antiviral activity results, coxsackie B2 (CXKB2), a major viral agent implicated in chronic myocarditis and potentially leading to congestive heart failure, exhibited significant sensitivity to the silydianin-rich extract across all three phases of the viral life cycle, as evaluated by both cytopathic effect (CPE) and qPCR-RT methods. The extract demonstrated the highest level of inhibition at all tested concentrations (200, 100, and 50 µg/mL) in the CPE assay. The findings from both methods were largely consistent, except at the 50 µg/mL concentration, where a slight discrepancy was observed. Specifically, at this concentration, the CPE assay showed approximately 80% inhibition, while qPCR-RT detected slightly higher viral RNA levels, with ΔC_t_ values of 0.83 ± 0.70 for virucidal activity and 1.33 ± 1.41 for cell protection relative to the virus control [7]. These results were compared with other studies that used natural products against coxsackievirus B2 (CXKB2). For instance, an EC_50_ of *Sesbania grandiflora* was evaluated in leaf and bark parts of the plant with various extraction solvents, including ethyl acetate, acetone, and methanol. The EC_50_ values ranged between 50 µg/mL and 200 µg/mL for acetone and ethyl acetate extracts, with no activity observed for methanol extracts [40]. Additionally, another study evaluated diterpenes from the roots of *Illicium jiadifengpi*, which demonstrated a strong EC_50_ for 19 compounds, achieving an EC_50_ of 2.71 µmol/mL with a high selectivity index of 90.37 [41]. These findings support our results, where the EC_50_ of the silydianin-rich extract against CXKB2 varied between 109.86 µg/mL and 169.40 µg/mL. For coxsackie A10 (CXKA10), a virus often associated with respiratory infections such as sore throat and associated HFMD [42], the virucidal assay showed complete viral replication, which was inconsistent with the cytopathic effect (CPE) assay results. In the CPE assay, inhibition was dose-dependent, reaching 80% at 200 µg/mL and 50% at 50 µg/mL. However, the cell protection and post-infection assays showed consistent results across both methods. This discrepancy may be due to the nature of the neutral red assay, which measures cell viability. Even if viral RNA is undetectable, indicating successful inhibition of viral replication, cells may still experience damage or stress from initial infection stages or factors like calpain-mediated cell death [7]. If the antiviral inhibits viral replication but does not fully prevent calpain activity or if the virus triggers calpain activation before full inhibition, the resulting cellular damage could lead to reduced viability in the colorimetric assay. A study on *Ganoderma neo-japonicum Imazeki* against CXKA10 and EV-A71 demonstrated its ability to inhibit viral replication effectively, even when treatment was administered at later stages post-infection [43]. On the other hand, while many natural products have been reported to have anti-EV-A71 activity, not all have shown broad-spectrum antiviral effects against both CXKA10 and CXKB2. Our inhibition results against EV-A71 demonstrated a good correlation between cytopathic effects and replication viral inhibition.

Poliomyelitis, a highly contagious viral disease, can cause irreversible paralysis within hours. Thanks to the global polio eradication initiative (GPEI), wild poliovirus types 2 and 3 have been eradicated, though type 1 remains endemic in Afghanistan and Pakistan [12]. Morocco was declared polio-free in 2015, but surveillance by the national polio laboratory of Morocco (NPLM) found 11 sabin-like vaccine strains over nine years, with 0.6% of residual paralysis cases linked to these attenuated strains in children under 5 years old [12]. In Morocco (2022), a tragic case involved a 4-year-old boy with acute flaccid paralysis (AFP) who likely developed vaccine-associated paralytic poliomyelitis (VAPP) and died 40 days after symptom onset. His contacts tested positive for sabin-like poliovirus Type 3, supporting the VAPP diagnosis [11]. While sabin-like poliovirus type 1 has a lower frequency of association with VAPP [44], antiviral activity is crucial in preventing such cases. Our study found that a silydianin-rich extract completely inhibited poliovirus RNA replication, with no detectable viral RNA and total inhibition of cytopathic effects across all phases of the viral cycle. This aligns with findings by Ionescu et al., where extracts of *Silybum marianum* and *Tamarix gallica* significantly reduced the infectivity of Poliovirus Type 1, LSc-2ab, after 60 min of contact, achieving lgTCID_50_ values of 5.5 and 6.2, respectively, compared with the virus control [45].

Gastroparesis is a gastric motility disorder characterized by delayed gastric emptying, where the stomach takes longer than normal to empty its contents into the small intestine [2]. This delay results from impaired muscle function in the stomach, leading to various non-specific digestive symptoms such as nausea, vomiting, bloating, early satiety, and abdominal pain [1]. Gastroparesis shares significant overlap with functional dyspepsia (FD), as both conditions exhibit similar pathophysiology and symptoms. The underlying pathology may be linked to dysbiosis, which is directly or indirectly associated with gut–brain axis alterations [2]. The etiology of gastroparesis remains poorly understood, with approximately 50% of cases classified as idiopathic, known as idiopathic gastroparesis (IGP). Other cases are associated with diabetes, post-surgical conditions, autoimmune diseases, and neurological disorders [2,3]. Enteroviruses, which infect epithelial cells in the gastrointestinal tract, have been implicated in some cases of IGP. Diagnosing idiopathic gastroparesis remains challenging, particularly as studies suggest a broad spectrum of infectious pathogens, especially a history of acute viral gastroenteritis, may be involved [3]. Research has increasingly focused on enteroviruses, suggesting that chronic enteroviral infection of the GI tract could contribute to the persistent symptoms of gastroparesis [3].

For instance, Barkin et al. studied patients diagnosed with IGP who were negative for other common causes of GP but showed delayed gastric emptying, with more than 10% of gastric contents retained at 4 h as measured by nuclear medicine gastric emptying scintigraphy. Among these patients, 82% were found to have active enterovirus infection based on gastric biopsies, and 89% of these patients responded to antiviral or immune therapies [3]. Another study detected double-stranded RNA in 63% of stomach biopsies from symptomatic patients using immunoperoxidase staining [46]. Enteroviral infections encompass a family of at least 71 small RNA viruses, including coxsackievirus groups A and B, echovirus, poliovirus, and others, without a universally effective treatment [47].

Given the lack of effective treatments for these viral strains, we conducted an in vivo study to assess the potential of our silydianin-rich extract in alleviating gastrointestinal disorders may be caused by enteroviruses, particularly focusing on improving delayed gastric emptying. Mice were pre-treated for seven days with the extract at doses of 200 mg/kg and 100 mg/kg. These doses align with standardized extract doses commonly used in human studies, where 100 to 200 mg taken orally twice daily falls within effective therapeutic ranges [48]. The results showed that at 100 mg/kg, gastric emptying was delayed compared to control mice, which received only physiological saline. In contrast, at 200 mg/kg, gastric emptying was significantly improved compared to both the 100 mg/kg group and the control group. Regarding gastrointestinal transit (GIT), there was a slight increase in the 200 mg/kg group compared to the control, while the 100 mg/kg group showed relatively unchanged results. However, these differences were not statistically significant. These findings contrast with a study by Illuri et al., which evaluated a combination of herbal extracts of *Ferula asafoetida* and *Silybum marianum* (known as Asdamarin). In that study, pre-treatment for one week in rats resulted in a significant increase in gastric emptying at doses of 50 mg/kg and 100 mg/kg, with percentages of 69.8% and 74.79%, respectively, which also correlated with a dose-dependent improvement in GIT [49]. The pharmacokinetic challenges of silybin and related silymarin flavonolignans, particularly their low solubility, poor absorption, and rapid metabolism, offer potential insights into the dose-dependent effects observed in our study on gastric emptying [50]. The oral bioavailability of silybin is extremely low, approximately 0.95% in rats [51], and even lower in humans, with only 0.45% excreted in the urine [52]. This limited bioavailability could explain the different effects observed at the 100 mg/kg and 200 mg/kg dosages in our study.

At 100 mg/kg, gastric emptying was delayed compared to the control group, likely because the dose was insufficient to reach therapeutic concentrations in the gastrointestinal system. The low bioavailability at this dose may have resulted in suboptimal effects, possibly interfering with normal physiological processes involved in gastric emptying. In contrast, at 200 mg/kg, the higher dose may have compensated for poor absorption, allowing more active flavonolignans to reach the target tissues within the gastrointestinal system, resulting in a therapeutic effect that improved gastric emptying.

The observed dose-dependent effects may be indicative of a biphasic response, where lower doses, like 100 mg/kg, relax gastric muscles, delaying emptying, while higher doses, such as 200 mg/kg, stimulate gastric activity, enhancing the emptying process. Additionally, the rapid phase II metabolism of silymarin, particularly its conjugation via glucuronidation in the intestine and liver, complicates its bioavailability, with only 10–17% remaining in its active, unconjugated form [50,53,54]. This may necessitate higher doses, such as 200 mg/kg, to reach sufficient pharmacologically active concentrations capable of improving gastric motility.

When comparing these findings to the study by Illuri et al., which used a combination of *Silybum marianum* and *Ferula asafoetida* (Asdamarin), the enhanced bioavailability in their study could be attributed to the synergistic effects of the combination. Our study, in contrast, focused solely on a silydianin-rich extract, which may not have had the same bioavailability benefits seen with multi-herbal formulations. These differences underscore the need for further research to elucidate the mechanisms underlying these dose-dependent effects and to optimize silymarin’s therapeutic potential for gastrointestinal disorders and antiviral activity, potentially through enhanced formulations that improve bioavailability.

## 4. Materials and Methods

### 4.1. Plant Material

In March 2022, the stems of *Silybum marianum* (milk thistle) were hand-harvested from open, minimally disturbed areas in the Chammaou neighborhood 34°03′49.0″ N 6°48′24.5″ W, in the city of Salé, located in the Rabat–Salé–Kénitra region of Morocco. These collection sites provided spacious, well-ventilated environments suitable for plant growth. The stems were identified by Prof. Hamid Khamar, a taxonomist at the Institute of Science, Mohammed V University, Rabat, Morocco. Following identification, the seeds were carefully separated from the stems by hand. The extracted seeds were then stored in a dark, dry place at a stable room temperature of 20 °C until further processing.

### 4.2. Extraction

The seeds were finely ground in a mortar of laboratory to a powder with a particle size ranging from 500 to 93 μm. From this powdered material, 50 g were accurately weighed and defatted with 500 mL of n-hexane through simple maceration to obtain a vegetable oil. After degreasing, the remaining material was soaked in methanol–water (80:20, *v*/*v*) in a screw-cap flask. The extraction mixture was gently stirred for 24 h at room temperature to enhance the extraction process. Subsequently, the mixture was filtered through Whatman No. 1 filter paper (GE Healthcare Bio-Sciences, Pittsburgh, PA, USA) to remove particulates. The filtrate was then concentrated under reduced pressure and lyophilized to eliminate any remaining solvents, and the resulting dry extract was stored at −20 °C until further analysis.

### 4.3. Biological Assay In Vitro

#### 4.3.1. Virus and Cell Line

The virus strains used in this experiment, including enterovirus (EV)-71, coxsackie B2, coxsackie A10, and poliovirus I (sabin-like type 1), were sourced from the national poliomyelitis laboratory at the national institute of health in Rabat, Morocco. These strains were originally isolated from the stools of children with acute flaccid paralysis. The RD cell line (Human Rhabdomyosarcoma Cells) is routinely used in this laboratory for the cultivation of viruses and the diagnosis of poliovirus and enterovirus infections. Cells (passage 3) were grown as an adherent monolayer in 75 cm^2^ flasks under standard conditions (37 °C ± 1 °C, 90% ± 5% humidity, and 5.0% ± 1% CO_2_/air) using a growth medium composed mainly of Dulbecco’s modified Eagle medium (DMEM) (Gibco, Vacaville, CA, USA) supplemented with 10% fetal bovine serum (FBS) (Gibco, USA), 1% penicillin–streptomycin (PSA) (Sigma Aldrich, St. Louis, MO, USA), 1% L-glutamine, and 1% essential amino acids. Virus propagation was conducted in a confluent monolayer of RD cells following a 1-h adsorption period of the virus inoculum at 37 °C in an incubator. After adsorption, the inoculum was removed and replaced with a maintenance medium consisting of DMEM with 2% FBS. The cytopathic effect (CPE) was monitored daily for up to 72 h using a phase-contrast microscope (EVOS XL Core). Viruses were harvested after three freeze–thaw cycles at −80 °C, followed by centrifugation at 14,000× *g* for 10 min at 4 °C to remove cell debris. The virus samples were then aliquoted and stored at −80 °C until further use.

#### 4.3.2. Titration of Viruses (TCID_50_)

Titration was monitored using the virus titer reduction method. Ten-fold dilutions of each stock virus, ranging from 10^−2^ to 10^−8^, were prepared in a medium consisting of DMEM without FBS. A volume of 100 µL from each dilution was added to 20 wells per dilution. Subsequently, 100 µL of a cell suspension containing approximately 1–2 × 10^5^ cells/mL was added to each well. The negative control wells contained RD cells without any virus. The plates were incubated under standard conditions (37 °C ± 1 °C, 90% ± 5% humidity, and 5.0% ± 1% CO_2_/air) and examined daily for the development of cytopathic effect (CPE) using an inverted microscope (EVOS XL Core) for 5–7 days. The virus titer was calculated using the Karber formula [55]:

Log TCID_50_ = L − d (S − 0.5)

where:

L = log of the lowest dilution used in the test.

d = difference between log dilution step.

S = dum of proportion of positive tests (i.e., cultures showing CPE).

#### 4.3.3. Cytotoxicity Assay

To evaluate the cytotoxicity of the hydro-methanolic extract, the colorimetric neutral red test was chosen using the method described in a study by Repetto et al. (2008) [56] with some optimizations. Neutral red (NR) is a weakly cationic dye that penetrates cell membranes by passive diffusion. It accumulates in lysosomes and binds to their matrix components via electrostatic and hydrophobic interactions. In brief, 2.105 cells/mL of RD cells were added to 96-well microtiter plates (200 µL per well) and incubated for 24 ± 2 h under standard conditions of 37 °C ± 1 °C, 90% ± 5% humidity, and 5.0% ± 1% CO_2_/air to obtain a monolayer of less than half confluence (<80%). The medium was then discarded from the plates, washed with 100 µL of pre-warmed D-PBS, treated with different concentrations (131.072, 163.84, 327.68, 409.6, 512, 640, 800, and 1000 µg/mL) of the plant extract and incubated for 48 ± 2 h under appropriate conditions. Two columns of monolayers of RD cells incubated with medium alone and with 1% DMSO were used as controls. A neutral red was prepared in DMEM at a concentration of 40 µg/mL and incubated overnight at the same temperature as the cells to allow the formation of NR crystals, indicating cell viability. The plate was rinsed again with pre-warmed PBS, and a volume of 200 µL of neutral red medium was added to each well and placed in the incubator for 4 h. After incubation, the NR medium was removed and the cells were rinsed with D-PBS before the NR desorb solution consisting of 50% ethanol (96%), 49% deionized water, and 1% glacial acetic acid (Sigma) was added to extract NR from the cells. After thorough shaking for 10 min in the shade of the stirrer, absorbance measurements were immediately performed at 540 nm ± 10 nm using a microplate reader. The experiment was repeated three times. CC_50_ was defined as the concentration of a compound at which 50% of cell viability was reduced. Cell viability was calculated using the following formula:

% Cell viability = (OD of Extract/OD of control cells) × 100

#### 4.3.4. Viral RNA Extraction and Quantitative Real-Time RT-PCR (rRT-PCR)

Total RNA was extracted from 140 µL of the culture supernatant of infected RD cells using the QIAamp Viral RNA Mini Kit (Qiagen, Santa Clarita, CA, USA), following the manufacturer’s instructions, and eluted with 40 µL of elution buffer. The detection of non-polio enteroviruses (EVs) and poliovirus I was performed using rRT-PCR One Step with primers targeting a conserved region of the 5′ untranslated region (5UTR) of the viruses. RNA samples were processed using the Invitrogen SuperScript™ III Platinum One-Step Quantitative kit (Thermo Fisher Scientific, Waltham, MA, USA) (Cat# 11732-020). The primer sequences were derived from Monpoeho et al. (BioTechniques 2000, 29, 88–93) [57], with the forward primer sequence being 5′-CCCCTGAATGCGGCTAATC-3′ (positions 451–469), the reverse primer sequence 5′-GATTGTCACCATAAGCAGC-3′ (positions 579–597), and the EV-probe sequence 5′-FAM-CGGAACCGACTACTTTGGGTGTCCGT-TAMRA-3′ (positions 532–557).

The reaction mixture was prepared by combining 10 µL of 2X RT-PCR mix, 1 µL of 10 µM forward primer, 1 µL of 10 µM reverse primer, 0.5 µL of 10 µM probe, 0.4 µL of enzyme mix, and 2.1 µL of water. Fifteen microliters of this mix were distributed into each well of a 100 µL plate suitable for the ABI 7500 Fast thermal cycler. Subsequently, 5 µL of the RNA sample was added to each well. The plate was sealed with an optical film and briefly centrifuged to ensure proper mixing.

The thermal cycling conditions on the ABI 7500 Fast thermal cycler were as follows: reverse transcription at 45 °C for 15 min, Taq inhibitor activation at 95 °C for 2 min, followed by 45 cycles of PCR amplification with denaturation at 95 °C for 15 s, and annealing/extension at 60 °C for 30 s. The total runtime for the thermal cycling was 82 min. Two independent experiments were conducted for each sample. The threshold cycle value (Cq) data were determined using default threshold settings, and the real-time RT-PCR was repeated two times. ΔCt values were calculated by subtracting the Ct values of virus control from the Ct values of virus samples treated with plant extracts.

#### 4.3.5. Antiviral Activity Profiles Using Neutral Red Assay and Quantitative Real-Time RT-PCR

##### Virucidal Activity

All viruses were pre-treated with four non-cytotoxic concentrations of the plant extract (400, 200, 100, and 50 µg/mL) at 100 TCID_50_/100 µL (TCID_50_ = 10−6.25) and incubated for 1 h at 37 °C ± 1 °C. Each virus strain was also incubated alone for 1 h as a control. RD cells, treated with trypsin, were seeded in 96-well culture plates at a density of 2 × 104 cells/well in a volume of 200 µL/well and incubated for 24 ± 2 h. Following this, the cells were infected with the pre-treated virus–extract mixture and the virus control and then incubated for 1 h at 37 °C ± 1 °C. After the incubation period, the inoculum was removed, and the cells were resuspended in maintenance medium (DMEM supplemented with 2% FBS). After a 24-h incubation, cell morphology was examined using an inverted microscope. The monolayers were examined microscopically to evaluate cytopathic effects (CPE), including rounding and other significant morphological changes relative to control cells (Figure 5) Culture supernatants were collected, and infectious viral titers were quantified using rRT-PCR. Cell viability was assessed using the neutral red assay as previously described.

##### Cell Protection Activity

Confluent RD cells in 96-well plates were pretreated with 100 µL of plant extract at concentrations of 400, 200, 100, and 50 µg/mL for 1 h at 37 °C ± 1 °C. The medium was then removed, and the wells were washed with pre-warmed PBS. Subsequently, the pretreated cells were inoculated with 100 µL of virus at a concentration of 100 TCID_50_ and incubated for 1 h at 37 °C ± 1 °C. For virus control, separate wells were inoculated with the virus without plant extract pretreatment and incubated under the same conditions. Following incubation, the viral inoculum was removed, the cells were washed with PBS, and maintenance medium (DMEM supplemented with 2% FBS) was added. The cells were then incubated for an additional 24 h. The culture supernatant was collected for rRT-PCR quantification of viral titers, and cell viability was assessed using the neutral red assay as previously described.

##### Post-Infection Activity

Confluent RD cells in 96-well plates were infected with a viral inoculum at a concentration of 100 TCID_50_/100 µL and incubated for 1 h at 37 °C ± 1 °C. After incubation, the viral inoculum was removed, and the wells were washed with pre-warmed PBS. A range of plant extract concentrations (400, 200, 100, and 50 µg/mL) diluted in DMEM supplemented with 2% FBS was added to the virus-infected cells at a volume of 200 µL per well. The cells were then incubated for 24 h. Subsequently, the culture supernatant was collected for rRT-PCR quantification of viral titers, and cell viability was assessed using the neutral red assay as previously described. The viral inhibition rate was determined using the following formula:

Inhibition Rate (%) = [(ODtv − ODvc)/(ODcc − ODvc)] × 100%

ODtv, ODvc, and ODcc indicate the absorbance of the test compounds in virus-infected cells, the absorbance of the virus control, and the absorbance of the cell control, respectively. The 50% effective concentration (EC_50_) was defined as the concentration that resulted in 50% protection of cells from viral infection.

### 4.4. Biological Assay In Vivo

#### 4.4.1. Animals

Adult male Swiss mice weighing approximately 25–30 g were obtained from the central animal care facility at the Faculty of Sciences, Mohamed V University, Rabat, Morocco. The mice were housed for at least 1 week in groups of five in propylene cages to acclimate to the laboratory conditions, which included a temperature of 25 ± 2 °C, relative humidity of 60–70%, and a 12-h light/dark cycle (lights on from 7:00 to 19:00). They had free access to standard laboratory feed and water. The animal experiments were conducted in the laboratory of animal physiology at the Faculty of Sciences, Rabat, Morocco, following the approval of the ethical committee of Mohammed V University in Rabat, under number PR-08-2024E.

#### 4.4.2. Determination of Gastric Emptying by Phenol Red Method

Gastric emptying in mice was measured using the method described by [58]. Eighteen adult male mice were divided into three groups of six animals each. Group I served as the control group and received physiological saline. Groups II and III were administered hydro-methanolic extract orally at doses of 100 mg/kg and 200 mg/kg, respectively, for one week. After an 18-h fasting period, the mice received their respective final doses. Two hours post-administration, a test meal comprising 1.5% carboxymethyl cellulose sodium salt containing 0.05% phenol red was orally administered. Twenty minutes after the test meal administration, the mice were euthanized using an overdose of ketamine/xylazine (80 mg/kg/20 mg/kg). The stomach was then excised for further analysis.

A separate group of four mice was used as a standard to determine the 100% phenol red content. These mice were sacrificed immediately after administration of the test meal to avoid errors due to stomach contractions during terminal convulsions [58]. The stomachs were rapidly ligated at the pylorus and cardia before removal. All stomach samples and their gastric contents were treated with 50 mL of 0.1 M NaOH, minced using scissors, and then centrifuged at 1800× *g* for 10 min. Moreover, 5 mL of the supernatant was precipitated with 0.5 mL of 20% trichloroacetic acid solution and centrifuged again at 1800× *g* for 20 min. Two milliliters of the resulting supernatant were realkalinized with 2 mL of a 0.5 M NaOH solution. The amount of phenol red in the supernatant was measured by absorbance at 570 nm using a microplate reader (Multiskan EX, Thermo Scientific, Waltham, MA, USA). Gastric emptying rate was calculated using the formula:

**Gastric emptying rate (%)** = (1 − amount of phenol red in the test sample/average amount of phenol red in the standard sample) × 100.

Gastrointestinal transit time (GIT) was calculated using the following formula:

**Gastrointestinal transit time (GIT)** = (Length of phenol red movement in the intestine/Total length of small intestine of rat) × 100.

### 4.5. Characterization of Silybum marianum Hydro-Methanolic Extract

The aqueous methanolic crude extract was diluted with water and then successively partitioned using ethyl acetate (EtOAc), chloroform (CHCl_3_), and *n*-butanol (*n*-BuOH) to isolate the primary compound responsible for the biological activity. This liquid–liquid partitioning efficiently facilitates the separation of the crude extract based on polarity, aiding in the isolation and identification of active components [59,60,61]. Nuclear Magnetic Resonance (NMR) spectroscopy was employed to elucidate the molecular structures of the compounds in the resulting fractions. Proton NMR (^1^H NMR, 600 MHz), Carbon-13 NMR (^13^C NMR, 151 MHz), DEPT 135 (Distortionless Enhancement by Polarization Transfer), and two-dimensional HSQC NMR analyses were conducted. The fractions were dissolved in deuterated DMSO (DMSO-d6), and spectra were recorded using high-field NMR equipment, Varian Direct-Drive 600 (1H 600 MHz/13C 150 MHz). The recorded spectra were then analyzed for chemical shifts expressed in δ (ppm) with reference to TMS and coupling constants (J) in hertz and integration using Mnova software (Mestrelab Research S.L.,version 14.2.1-27684, Santiago de Compostela, Spain, 2021). Peaks were assigned by comparison with literature data, aiding in the identification and structural elucidation of the compounds. Additionally, accurate mass determinations were performed on a SYNAPT G2-Si Q-TOF mass spectrometer (Waters, Milford, MA, USA) equipped with high-efficiency T-Wave ion mobility and an orthogonal Z–spray™ electrospray ionization (ESI) source. MassLynx v.4.1 software was used for HRMS instrument control, peak detection, and integration.

### 4.6. Statistical Analysis

The data were analyzed using GraphPad Prism version 9.0, with values reported as mean ± S.E.M. Statistical analysis was performed using one-way ANOVA followed by the Tukey test, with significance set at *p* < 0.05. Regression analysis was applied to determine EC50 and CC50 by converting values to logarithmic form and normalizing the data. Statistical significance levels were indicated as * *p* < 0.05, ** *p* < 0.01, *** *p* < 0.001, and **** *p* < 0.0001.

## 5. Conclusions

In conclusion, our study demonstrates the significant antiviral activity of a silydianin-rich extract against coxsackievirus B2, coxsackievirus A10, poliovirus SL-1, and enterovirus EV71, highlighting its potential for further investigation. The extract also shows promise in alleviating gastrointestinal disorders that could be related to enteroviral infections, particularly by improving delayed gastric emptying. Although the improvements were not dose-dependent, this suggests a need for further research into the mechanism of action of silydianin. As silydianin has been less studied than silybin and its isomers, further exploration of its purified form could offer new treatment options for enteroviral infections and associated conditions, such as acute flaccid paralysis and hand–foot–mouth disease, as well as help mitigate persistent symptoms from previous enteroviral infections affecting the gastrointestinal system.

## Figures and Tables

**Table 1:** The effective concentration 50% (EC_50_) and selectivity index (SI) of *Silybum marianum* extract on studied enteroviruses.

Infections Assay	Virus							
	Coxsackie B2		Coxsackie A10		EA71		Poliovirus SL-1	
	^a^EC_50_ (µg/mL)	^b^SI	^a^EC_50_ (µg/mL)	^b^SI	^a^EC_50_ (µg/mL)	^b^SI	^a^EC_50_ (µg/mL)	^b^SI
Virucidal activity	109.86 ± 29.04	3.73	112.40 ± 20.36	3.64	100.31 ± 2.70	4.08	110.78 ± 13.90	3.69
Cell protection	169.26 ± 41.61	2.42	120.88 ± 43.62	3.39	186.100 ± 45.47	2.20	173.36 ± 54.33	2.36
Post-infection	169.40 ± 58.76	2.41	105.06 ± 1.96	3.90	107.67 ± 6.04	3.82	120.96 ± 19.92	3.38
CC_50_ (µg/mL)	409.80 ± 33.70							

^a^EC_50_ refers to the concentration required to inhibit 50% of the viral activity, whereas ^b^SI (selectivity index) is calculated as the ratio of CC_50_ to EC_50_.

**Table 2:** ΔC_t_ values determined using the real-time RT-PCR assay. RD cells were infected with four enteroviruses and incubated with treatment doses of the plant extract at 37 °C for 48 h. Viral RNA was extracted from 100 µL of culture supernatant and subjected to rRT-PCR. Inhibition was assessed by comparing C_t_ values of treated samples to the control using the formula ΔC_t_ treated = C_t_ treated − C_t_ control. Data are expressed as mean ± SD from two independent experiments. Statistically significant differences are indicated by different letters (a, b) for *p* < 0.05, with ns denoting non-significant differences, as determined by the *t*-test.

Infections Assays	Concentraions (µg/mL)	ΔC_t_ Values			
		CXKB2 Virus	EA71 Virus	CXKA10 Virus	Polio Virus-SL1
Virucidal activity	200	-	14.167 ± 0.707 ^b^	-	-
	100	8.833 ±0.707 ^a^	13.167 ± 0.707 ^a^	-	-
	50	0.833 ± 0.707 ^a^	9.667 ± 1.414 ^ab^	-	-
Cell protection	200	8.333 ± 1.414 ^ns^	3.667 ± 1.414 ^b^	-	-
	100	5.333 ± 1.414 ^a^	3.667 ± 1.414 ^a^	-	-
	50	1.333 ± 1.414 ^a^	1.167 ± 0.707 ^ab^	2.167 ± 0.707	-
Post-infection	200	-	6.667 ± 2.828 ^b^	3.667 ± 1.414 ^ns^	-
	100	8.833 ± 0.707 ^a^	4.167 ± 2.121 ^a^	3.167 ± 0.707 ^ns^	-
	50	2.333 ± 2.828 ^a^	1.167 ± 2.121 ^a^	1.767 ± 1.414 ^ns^	-

## Data Availability

The datasets supporting the results of this article are included within the article itself.
